# Effectiveness and cost-effectiveness of out-of-hours palliative care: a systematic review

**DOI:** 10.12688/hrbopenres.13006.1

**Published:** 2020-03-13

**Authors:** Bridget M. Johnston, Rachel McCauley, Regina McQuillan, Mary Rabbitte, Caitriona Honohan, David Mockler, Steve Thomas, Peter May

**Affiliations:** 1 Centre for Health Policy and Management, Trinity College Dublin, University of Dublin, Dublin, D2, Ireland; 2 Palliative Medicine, St Francis Hospice, Dublin, D05 T9K8, Ireland; 3 Palliative Medicine, Beaumont Hospital, Dublin, D09 V2N0, Ireland; 4 All-Ireland Institute of Hospice and Palliative Medicine, Dublin, D6W, Ireland; 5 The Library of Trinity College Dublin, Trinity College Dublin, University of Dublin, Dublin, D2, Ireland; 6 The Irish Longitudinal study on Ageing (TILDA), Trinity College Dublin, University of Dublin, Dublin, D2, Ireland

**Keywords:** Palliative Care, Terminal Care, Health Care Quality, Health Care Access, Health Care Evaluation, After-Hours Care, Systematic Review

## Abstract

**Background:** Out-of-hours palliative care is a priority for patients, caregivers and policymakers. Approximately three quarters of the week occurs outside of typical working hours, and the need for support in care of serious and terminal illness during these times is commonplace. Evidence on relevant interventions is unclear.

**Aim:** To review systematically the evidence on the effect of out-of-hours specialist or generalist palliative care for adults on patient and caregiver outcomes, and costs and cost-effectiveness.

**Methods:** A systematic review of peer-reviewed and grey literature was conducted. We searched Embase, MEDLINE [Ovid], Cochrane Library, CINAHL, Allied and Complementary Medicine [Ovid], PsycINFO, Web of Science, Scopus, EconLit (Ovid), and grey literature published between 1 January 2000 and 12 ^th^ November 2019. Studies that comparatively evaluated the effect of out-of-hours specialist or generalist palliative care for adults on patient and caregiver outcomes, and on costs and cost-effectiveness were eligible, irrespective of design. Only English-language studies were eligible. Two reviewers independently examined the returned studies at each stage (title and abstract review, full-text review, and quality assessment).

**Results:** We identified one eligible peer-reviewed study, judged as insufficient quality. Other sources returned no eligible material. The systematic review therefore included no studies.

**Conclusions:** The importance of integrated, 24-hour care for people in line with a palliative care approach is not reflected in the literature, which lacks evidence on the effects of interventions provided outside typical working hours.

**Registration:** PROSPERO CRD42018111041.

## Introduction

### Background

Demographic ageing brings a growing burden of complex and life-limiting disease, posing a major challenge for health systems worldwide ^1, 2^. The value of and need for an integrated palliative care approach to improve quality of life for people with life-limiting illness and their families is widely recognised ^3– 6^.

While preferences vary by conditions and personal circumstances, studies routinely find that a majority of people with terminal illness prefer to stay at home, provided that they can access appropriate services and supports ^7– 12^. Typical professional hours run from 9.00am to 5.00pm, Monday to Friday, or similar, meaning that three quarters of a patient’s week occurs ‘out of hours’. Meeting the policy goal of universal palliative care available according to need therefore requires comprehensive out-of-hours care provision. Poor patient outcomes have been linked to inadequate community supports and a lack of patient confidence in out-of-hours access ^13, 14^.

In the United Kingdom, guidelines from the National Institute for Clinical Excellence have recommended 24-hour specialist palliative care access for people with cancer since 2004 ^15^. Nevertheless, priority-setting exercises with patients, carers, volunteers, and health and social care professionals have identified “the best ways of providing palliative care outside of working hours” as the top research priority in both the United Kingdom and Ireland ^16, 17^.

### Policy context

Ireland was among the first countries in the world to adopt a national palliative care policy in 2001 ^18, 19^. Policymakers have initiated an update of the policy, to start in 2020 ^20^, which will be conducted within the Sláintecare agenda ^21^. Among multiple goals, Sláintecare aims to reorient the Irish health and social care system away from the acute setting to community-based services ^22^. In this context of system-level reform and palliative care policy review, the Irish Department of Health funded this study to establish best available international evidence on out-of-hours palliative care.

Palliative care is an interdisciplinary field that occurs across settings and cross-cuts many other medical specialisms. Out-of-hours services are therefore likely to be diverse, potentially including the further extension of existing services, e.g. a specialist inpatient team working evenings and/or weekends; development of new services, e.g. telehealth services offering symptom advice or counselling; or the upskilling of other branches of health care, e.g. palliative care education for emergency department triage nurses, general practitioners, or paramedics.

Development of out-of-hours services should be guided by high-quality evidence on best practice. This could include studies evaluating a range of important considerations such as patient and public preferences, population need, workforce planning, commissioning and approaches to implementation and integration of services. However, making policy recommendations requires evidence about their effectiveness or cost-effectiveness.

### Rationale

Multiple systematic reviews have been reported in the field of palliative care. Some have examined effectiveness and/or cost-effectiveness across multiple settings and configurations ^23– 33^, while others have focused on specific models and settings, including hospital inpatient ^34, 35^ and outpatient ^36^, home care ^37^, and day care settings ^38^, as well as care provided by unpaid family carers ^39^. However, we were not aware of any review identifying and organising systematically the evidence on out-of-hours services.

We therefore conducted a systematic review of peer-reviewed studies and grey literature that specifically addressed this research question:

What is the effect of out-of-hours specialist and generalist palliative care services on patient and family/caregiver outcomes, and on costs and cost-effectiveness?

Studies that comparatively evaluated the effect of out-of-hours specialist or generalist palliative care for adults on patient and caregiver outcomes, and on effectiveness and cost-effectiveness were eligible, irrespective of design.

## Methods

### Protocol and registration

We registered the review protocol on PROSPERO CRD42018111041 ^40^.

### Eligibility criteria

Studies reporting on the following PICOS (Participants, Interventions, Comparators, Outcomes, and Study design) were eligible for this review.

***Types of participants.*** Studies were eligible if they examined adults (18 years and over) in their last year of life and/or had a terminal illness and/or had other serious/complex medical needs, and/or were a carer for someone who met these criteria.

We placed no restrictions on diagnosis/need, or on patient/carer perspective. Studies of children (under 18 years of age) and studies pooling children and adults without reporting the results separately were ineligible.

***Types of interventions/exposure/comparators.*** Out of hours was defined as outside of typical working hours (i.e. 9.00am–5.00pm, Monday to Friday) and therefore including overnight, weekends, and public holidays. In the event of any ambiguities with respect to intervention timing in otherwise eligible studies, we decided prior to beginning our review to resolve these through discussion and consensus among the core researchers of the review team, and, where appropriate, through contact with the author of the study in question.

The best-established models of care internationally are inpatient hospices, inpatient and outpatient hospitals, home care, and day care. We did not define the term ‘palliative care’ before starting our review, seeking studies of out-of-hours care for people with life-limiting illnesses, resolving ambiguities through discussion and consensus among the review team, and, where appropriate, contacting the corresponding author. We did not restrict eligibility by either setting or generalist/specialist configuration of staff.

Eligible interventions therefore included both new models of care provided outside of typical working hours, and already established models of care where the effect of out-of-hours provision specifically was evaluated and reported on.

We required that studies were comparative in nature. Eligible comparators therefore included usual care and/or alternative models of out-of-hours palliative care.

***Types of outcomes.*** Our primary outcomes of interest were the effectiveness (patient/carer outcomes) and cost-effectiveness (economic outcomes) of interventions.

We took a broad approach to all outcomes. Patient and/or carer outcomes could be quantitative or qualitative, and included quality of life and experience, as well as survival. Effectiveness outcomes had to be reported by the patients, carers, or a credible proxy. Perceptions of patient and/or carer outcomes from healthcare staff or administrators were not deemed eligible. Eligible economic outcomes included any resource utilisation typically considered to fall within the societal viewpoint (e.g. cost to payers, service users, and families, as well as unpaid care). We did not require resources to represent the literal cost of provision, but also classed non-cost measures of resource utilisation as eligible – e.g. insurance programme charges and frequency use data combined with validated unit costs. Any study reporting within cost-consequence frameworks, such as cost-effectiveness analysis and cost-utility analysis, was also eligible.

Ineligible outcomes were those that related to other parties, e.g. the experience or capacity of clinical staff or the perceptions of healthcare workers of the effectiveness and cost-effectiveness of services.

***Types of studies/reports.*** Study designs vary considerably in research on serious illness in the context of profound ethical and practical considerations ^41^. As such, we did not restrict our search to any one design. We planned to include the following types of studies: prospective/retrospective cohort studies, before-and-after studies, randomised controlled trials, economic evaluations, qualitative/descriptive studies, and pilot studies.

We excluded studies that did not comparatively measure the effect of interventions on our outcomes of interest, since comparative evaluation was considered intrinsic to our research question.

***Time period.*** Studies were only eligible if they finished data collection no earlier than 1 January 1996 and were published no earlier than 1 January 2000.

We based this decision both on the rapidly changing palliative care landscape in the period 1998–2018, and on our own national context: Ireland’s current official policy was written in 2001 (and therefore had the chance to incorporate relevant research prior to that point).

***Cultural and linguistic range.*** Given the skills of the research team, only English-language materials were eligible for inclusion.

All returned studies in a language other than English were recorded and are reported separately in our results.

### Database search: information sources and search terms

Two information specialists (CH and DM) searched the following electronic databases:

- Embase
- MEDLINE (Ovid)
- Cochrane Library
- CINAHL
- Allied and Complementary Medicine (Ovid)
- PsycINFO
- Web of Science
- Scopus
- EconLit

Searches were conducted on November 12 ^th^, 2019.

Information specialists (CH and DM) and subject experts (BMJ, PM, RMcQ, MR) devised searches for keywords in the titles, abstracts, subject headings, and controlled vocabulary of the databases ( Table 1). We searched only for articles published from 1 January 2000 onwards, in line with our eligibility criteria.

**Table 1.** Database search terms (example using Embase).

#	Search terms
1	‘palliative therapy’/exp OR ‘terminal care’/exp OR ‘terminally ill patient’/exp OR ‘hospice’/exp
2	Palliat*:ti,ab
3	((terminal* OR hospice* OR ‘end-of-life’ OR ‘end-stage’ OR ‘last year of life’ OR LYOL OR ‘life’s end’) NEAR/5 (care OR caring)):ab,ti
4	((advanced OR terminal*) NEAR/5 (ill* OR disease*)):ti,ab
5	(‘end stage’ OR ‘end of life’ OR ‘last year of life’ OR LYOL or ‘life’s end’):ti,ab
6	#1 OR #2 OR #3 OR #4 OR #5
7	‘OOH care’/exp
8	(‘after-hour*’ OR ‘OOH’ OR ‘outside normal hours’ OR ‘out of office hours’ OR ‘outside office hours’ OR ‘after office hours’ OR ‘outside normal working hours’ OR weekend* OR holiday* OR ‘off-hour*’):ti,ab
9	#7 OR #8
10	#6 AND #9

### Grey literature search: information sources and search terms

Two information specialists (CH and DM) searched the following grey literature sources:

- Google Scholar
- OpenGrey
- ClinicalTrials.gov
- World Health Organization International Clinical Trials Registry Platform (WHO ICTRP)
- ProQuest Dissertations & Theses (United Kingdom [UK] and Ireland)
- RIAN.ie
- Lenus
- EThOS

Searches were conducted on November 12 ^th^, 2019. Keywords from the database search were applied ( Table 2).

**Table 2.** Grey literature search terms (example using OpenGrey).

#	Search terms
1	(palliative OR “terminal care” OR “terminally ill” OR hospice* OR “end-of-life” OR “end-stage” OR “last year of life” OR LYOL OR “life’s end”) AND (“after-hours” OR “OOH” OR “outside normal hours” OR “out of office hours” OR “outside office hours” OR weekend* OR holiday* OR “off-hour*”)
2	Palliative hours
3	#1 OR #2

### Other sources

We decided at the outset that all studies found to be eligible and passing quality assessment would be reviewed for references to other potentially relevant studies.

We also checked other systematic reviews for citations of relevant studies: We checked all studies included in all systematic reviews returned by our database search, and all studies included in 17 other reviews that we knew at the outset and covered all major palliative care settings ^23– 39^.

### Study selection

***Screening of titles and abstracts.*** Two information specialists (CH and DM) executed the searches and made the retrieved citations available in EndNote. Two team members (BMJ and PM) uploaded these citations to the online reviewer tool Covidence and reviewed titles and abstracts independently using the eligibility criteria described. Conflicts between the two reviewers were resolved using discussion and consensus.

***Screening of full-text reports.*** Two team members (BMJ and PM) independently reviewed all studies that were advanced to full-text screening on Covidence using the eligibility criteria described above. Conflicts between the two reviewers were resolved using discussion and consensus.

***Assessment of methodological quality/bias.*** Following agreement on eligibility, each study was assessed for methodological quality using one of a number of standardised instruments developed by the Critical Appraisal Skills Programme ( CASP), which also provides recommendations for exclusion of studies. Since multiple study designs were eligible, we decided prior to data collection to use the specific CASP tool most appropriate to each study (e.g. the CASP Case Control Study Checklist, the CASP Economic Evaluation Checklist, the CASP Qualitative Checklist, and so forth).

Two team members (BMJ and PM) quality-assessed all eligible studies independently. Conflicts between the two reviewers were resolved using discussion and consensus.

***Other sources.*** Two information specialists (CH and DM) compiled all returned grey literature in EndNote. For Google Scholar, the first 10 pages (100 items) were collated; for all other sources, all returned items were collated. One team member (RMcC or PM) reviewed each grey literature item for potential relevance to this review. Studies published in the peer-reviewed literature were discarded if they had already been returned by the database search. Where the reviewer was uncertain of relevance, s/he conferred with another team member (BMJ).

One team member (PM) performed the review of the reference lists of other systematic reviews.

### Data collection process

Two team members (BMJ and PM) were to extract data independently. Conflicts between the two reviewers would be resolved using discussion and consensus.

### Data items

We decided prior to data collection to extract the following data items from studies that were eligible and of sufficient quality: design (e.g. randomised controlled trial, prospective cohort study, case-control study, etc.); country of origin; care setting; model of care; level(s) of provider expertise/training; sample size; patient characteristics; carer characteristics; recruitment and sampling; ethical issues, including consent; research question; outcomes; approach to confounding; statistical methods; and findings.

### Synthesis of results

We decided to perform meta-analyses of included studies where possible due to homogeneity of methods, participants, interventions, and reporting. Given the wide range of outcomes of interest, it was not possible to specify all outcome measures or synthesis methods prior to data collection.

### Prior versions of this work

An earlier version of this systematic review, along with other components of the HRB/DOH-funded project, were previously published on the HRB website ^42^. The only substantive change to the review between versions is that we updated the search strategy from August 2018 to November 2019. No new studies of relevance were identified.

An oral presentation of this project was made at the SPHeRE Network 5th Annual Conference, Dublin, February 2019. A poster presentation was made at the 16 ^th^ World Congress, European Association for Palliative Care, Berlin, May 2019.

## Results

### Database search

**Figure 1.**
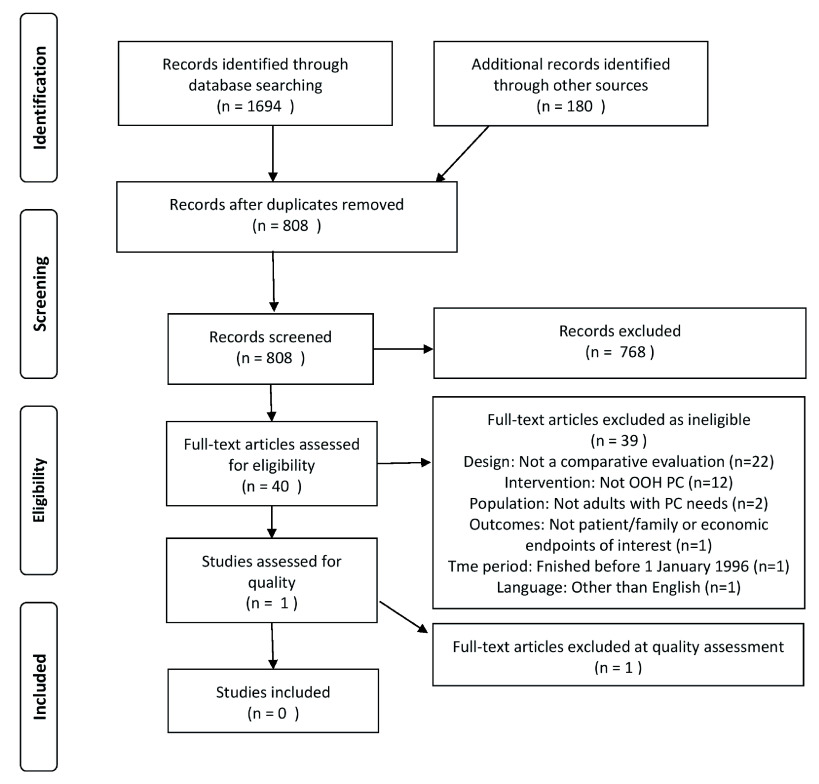
PRISMA diagram: database search, grey literature review and study selection.

Our search of nine databases is summarised in Figure 1.

The search yielded 1,694 citations, of which 958 were duplicates. We reviewed the remaining 736 unique titles/abstracts, of which 696 did not meet the eligibility criteria. One paper was excluded at this stage due to not being in the English language; it was in Dutch ^43^.

We then reviewed the remaining 40 full texts, of which one met the eligibility criteria ^44^.

We reviewed this study using the cohort study checklist. Under this checklist, the first section is ‘Section A: Are the results of the study valid?’. Section A consists of two questions:

1. Did the study address a clearly focused issue (e.g. population studied, risk factors, outcomes)?
2. Was the cohort recruited in an acceptable way (e.g. representativeness, criteria, exhaustiveness)?

Two independent reviewers concluded that the answer to each question was ‘No’. Specifically, reviewers recorded concerns about how the comparison group were identified. The authors do not provide eligibility criteria (Question 1) or methods of recruitment/identification (Question 2). The study was therefore not advanced to full CASP assessment.

The database search therefore identified zero peer-reviewed studies evaluating the impact of out-of-hours palliative care for adults on patient/carer outcomes and/or economic outcomes.

### Other sources

Our search of grey literature sources yielded 180 items, of which 108 were peer-reviewed articles already returned by the database search. The remaining 72 items were reviewed for duplicates, which were discarded (n=4). None of the remaining 68 items were found to contain evaluations relevant to this systematic review.

Our review of the citations found in other systematic reviews examined one published systematic review returned by our database search ^45^, and from 17 other reviews that we were already aware of ^23– 39^. No additional eligible papers were identified.

## Discussion

### Main results

We identified no study of sufficient quality that evaluated the effectiveness or cost-effectiveness of out-of-hours specialist or generalist palliative care for adults.

The reasons for this lack of evidence are important to understand if future research is to address an established priority for patients, carers, volunteers and professionals. Some reasons may be generic within palliative and end-of-life care. Primary research studies face practical and ethical challenges, resulting in a small evidence base in all settings relative to policy relevance ^31, 33^. The relative newness of palliative care as a specialism is also a potential factor. For example, medical research activity is highest in the United States, where palliative care is heavily hospital-based and hospital costs are a major public policy issue ^46^. Yet a systematic search of the literature to 2018 identified only one prospective economic study of this model of care ^35^. Since out-of-hours services are new even within this relatively young field, it is perhaps not surprising that research is underdeveloped.

Other reasons may be specific to out-of-hours services. From an overall service perspective, out-of-hours is one component of a model of care ^47^. Where out-of-hours care is one element of the model under evaluation, it may not be identified specifically in reporting under keywords, MeSH terms or abstracts. There is therefore a risk of under-identification in our methods. However, any study isolating the effect of out-of-hours within a wider evaluation of palliative care provision was eligible for our review, and any study evaluating that specific effect would likely report this in the abstract. Studies most likely to go unidentified by our review are those where a service is provided on a 24-hour basis, and the out-of-hours elements were not separated in analysis. In this case, the study would not have been eligible under our criteria in any case.

Finally, it is important to note that our findings do not mean that no literature on out-of-hours palliative care exists, simply that evaluations specifically were not identified. Our review identified, but did not include as eligible, topics including general practitioner perspectives on out-of-hours palliative care ^48, 49^; pilot programmes on, inter alia, prescribing and telehealth ^50, 51^; and an ongoing systematic review to identify quality improvement projects in out-of-hours palliative care ^52^. These studies and others advance understanding outside of our evaluative focus, as well as potentially improving future evaluative studies.

### Strengths and limitations

Any systematic review is vulnerable to missing relevant material, either through mis-specification of search terms or errors in review. We minimised these risks by establishing clear PICOS and eligibility criteria prior to data collection, employing a combination of subject and information specialists in executing searches, using two reviewers independently throughout the process, and examining studies that were included in multiple other prior reviews of palliative care in different settings.

We excluded studies from prior to 2000, which in principle may have excluded relevant evidence. Time cut-offs are inherently arbitrary but in this context were deemed important, palliative care practice from over two decades ago being very different to today in all countries. Specifically, this project was commissioned by Irish policymakers ahead of a review of national policy ^20^; prior policy was published in 2001 meaning that all relevant evidence to that point ought to have been collated already and so we established 2000 as an obvious cut point. As noted in Figure 1, one study was excluded due to the time criterion ^53^. We excluded one study due to our English language criterion ^43^.

### What this study adds

This study illustrates that while out-of-hours palliative care is a recognised priority for patients and policymakers, no evidence base exists on which services are beneficial for patients and worthy of health care funding.

The lack of evidence underscores the need for future studies to incorporate measurement of the effectiveness and/or cost-effectiveness of out-of-hours services. In principle there are two ways that such evaluations might be initiated. First, data are already collected by statutory bodies and other providers on existing out-of-hours services. Appropriate analyses of these data could produce the sort of evidence that this review hoped to identify, albeit statutory data tend to focus more on process than outcomes, which limits analytic scope ^19^.

Second, original research must be conducted to collect data and evaluate out-of-hours care across its multitude of settings and practitioners. Consistent with other areas of palliative and end-of-life care research, this agenda will have to be flexible and pragmatic in matching methodological approaches to specific problems ^41^.

### Conclusion

We conducted a systematic review of peer-reviewed and grey literature in order to identify evidence on the impact of out-of-hours palliative care for adults on patient and carer outcomes, and on economic outcomes.

We searched nine databases using both information and subject specialists, and we searched grey literature, including doctoral theses and policy repositories. Our database search yielded only one relevant study, which two independent reviewers judged to be of insufficient quality to include in the review. Our search of other sources found no relevant material.

The evidence base on out-of-hours palliative care is very small relative to importance to patients and policymakers. These evidence gaps must be urgently addressed.

## Data availability

### Underlying data

All data underlying the results are available as part of the article and no additional source data are required.

### Reporting guidelines

Open Science Framework: Appendix to: [Effectiveness and cost-effectiveness of out-of-hours palliative care: a systematic review]. https://doi.org/10.17605/OSF.IO/6EP9A ^54^.

Data are available under the terms of the Creative Commons Zero “No rights reserved” data waiver (CC0 1.0 Public domain dedication).

## Disclaimer

This work does not represent the opinions of the DOH or the HRB, and any errors or omissions are the responsibility of the authors.
