# Supplementary material for: Effectiveness and cost-effectiveness of out-of-hours palliative care: a systematic review
Source: HRB Open Res. 2020 Mar 13;3:9. [Version 1] doi: 10.12688/hrbopenres.13006.1 (PMC7845148; doi:10.12688/hrbopenres.13006.1)
Supplement: Peer review history

# Peer review: Reviewer response for version 1

**DOI:** 10.21956/hrbopenres.14096.r28721

**Published:** 2021-02-02

**Authors:** Melissa J. Bloomer

**Affiliations:** 1 School of Nursing and Midwifery, Deakin University, Geelong, Australia; 2 Institute for Health Transformation, Deakin University, Geelong, Australia

The focus of this systematic review was to examine evidence of the effect of out-of-hours palliative care on adults patients and or their caregivers. Whilst no studies were found for inclusion in this review, on the whole, the methods of the systematic review were sound.

 In terms of the inclusion criteria, given the focus was specifically on out-of-hours palliative care services. Hence further justification of why patients with serious/complex medical needs were included in the description of types of participants would be beneficial. This is because as a descriptor, ‘serious/complex medical needs’ could apply to persons were not approach end of life, such as those recently discharged after surviving critical illness, for whom other services such as critical care outreach may be available. I would also suggest that to define 'out-of-hours' as 9.00am to 5.00pm Monday to Friday may also be a factor in limiting the number of eligible papers. For example, a specialist palliative care service operating into the evenings and seven days per week , and therefore not labelled as ‘out-of-hours', would not have met the inclusion criteria.

 In the Discussion, the authors highlight that one reason for the lack of papers eligible for inclusion in this review is that out-of-hours care may be just one element of a palliative care service, and data pertaining to out-of-hours service provision may not be reported as a primary outcome, but rather as a secondary or minor finding. In this case, reference to out-of-hours services may not feature in keywords, the title or abstract, limiting retrieval in a database search. 

 It stands to reason however, that there are likely other factors contributing to the dearth of eligible studies about out-of-hours palliative care. Given that a recent case review of a Hospice at Home service in the UK demonstrated that the increasing demand for the service outstripped supply ^1^, I would encourage the authors to consider alternate sources of out-of-hours support for patients with palliative care needs and their carers. For example, patients (and or family caregivers) may seek similar support or care from primary care providers such as their general practitioner or primary treating physician, district nurses and/or an emergency department ^2^. For this reason, inclusion of any service that provides out-of-hours care to patients with palliative care needs may have improved the search outcome.

Are the rationale for, and objectives of, the Systematic Review clearly stated?

Yes

Is the statistical analysis and its interpretation appropriate?

Not applicable

Are sufficient details of the methods and analysis provided to allow replication by others?

Yes

Are the conclusions drawn adequately supported by the results presented in the review?

Yes

Reviewer Expertise:

End-of-Life Care; Palliative Care; Acute Hospitals.

I confirm that I have read this submission and believe that I have an appropriate level of expertise to confirm that it is of an acceptable scientific standard.

## References

[1] : Persistent inequalities in Hospice at Home provision. *BMJ Support Palliat Care*.2020;10(3) : 10.1136/bmjspcare-2017-001367 e23 10.1136/bmjspcare-2017-001367 29444775PMC7456670

[2] : *Am J Hosp Palliat Care*.2017;34(10) : 10.1177/1049909116676774 984-990 10.1177/1049909116676774 27903774

# Peer review: Reviewer response for version 1

**DOI:** 10.21956/hrbopenres.14096.r28631

**Published:** 2021-01-27

**Authors:** Jackie Robinson

**Affiliations:** 1 School of Nursing, University of Auckland, Auckland, New Zealand

This review focuses on an important part of how palliative care is provided in the setting of out of hours care. The review is very well presented and provides comprehensive explanations on all aspects of the methodology adopted providing a clear protocol for future reviews on the topic.

 The review has highlighted a significant deficit in the literature about the efficacy and cost effectiveness of out of hours palliative care. The authors have highlighted a number of limitations which may have impacted on the identification of relevant studies. Extending the search terms to include studies reviewing models of care which are inclusive of the provision of out of hours care might have revealed some information that would be useful. In addition, limiting studies to those which use comparative measures on the effect of interventions was a major limitation in identifying articles that may be of interest. For example, reviewing the literature on patient and family’s experiences of different models of out of hours care may have provided valuable information to inform policy recommendations. models. This is especially important given the ethics of carrying out comparative studies in palliative care.

 Recommendations for future research in this area of palliative care are provided. Given the increasing focus by policy makers and leaders in palliative care to provide equitable access to appropriate palliative care, this is an important area of research.

Are the rationale for, and objectives of, the Systematic Review clearly stated?

Yes

Is the statistical analysis and its interpretation appropriate?

Yes

Are sufficient details of the methods and analysis provided to allow replication by others?

Yes

Are the conclusions drawn adequately supported by the results presented in the review?

Yes

Reviewer Expertise:

My area of research is in palliative and end of life care with a specific focus on inequities across care settings and population groups.

I confirm that I have read this submission and believe that I have an appropriate level of expertise to confirm that it is of an acceptable scientific standard.

# Peer review: Reviewer response for version 1

**DOI:** 10.21956/hrbopenres.14096.r27708

**Published:** 2020-07-24

**Authors:** Bridget Candy

**Affiliations:** 1 Marie Curie Palliative Care Research Department, Division of Psychiatry, University College London, London, UK

This review focuses on a priority topic, namely how best to provide palliative support for the three-quarters of each week which are outside of office hours. The review is well and thoughtfully written and rigorous in its conduct.

 It is shame the review did not find any relevant studies to include and thereby could not give any evidence informed recommendations. The authors have in their discussion pointed out reasons for not finding any evidence. The first I listed as I reviewed this paper and for which the authors discuss was limitations in the search terms. In that a model of care may not highlight as a key word or descriptive that it provided an/was an out of hours service. Can the authors comment on whether if they did update this search if they would consider extending their criteria to include known models of care which while provide 24/7 care are not necessarily labelled as out of hours care? There is also the issue of ethics and comparison that challenges as the authors state conducting a comparative study in this area. How can you provide extra care to some and not to others. So then the question is what would be the best comparator group? The authors were limited to papers only in English. However from the English abstract of the Dutch paper they excluded, based on being non English, it would seem that this paper may have something to add to this review, even if it is just in the discussion. This is in regards to the halting of their study because of the additional costs of 'out of hours' and their finding of no clear benefit. I wonder if the Dutch authors have an English translation of the paper or a Dutch colleague could assist in translating it?

 I also wonder if a 'lower' level review is warranted. That is one that maps out what types of models of care there are that involve out of hours support. Such a review using some kind of systematic framework could explore other ways to see what may work and in what context. Such an endeavour may give some clearer direction for future research. Thereby giving some evidence informed recommendations which may lead following further evaluation to policy recommendations.

Are the rationale for, and objectives of, the Systematic Review clearly stated?

Yes

Is the statistical analysis and its interpretation appropriate?

Not applicable

Are sufficient details of the methods and analysis provided to allow replication by others?

Yes

Are the conclusions drawn adequately supported by the results presented in the review?

Yes

Reviewer Expertise:

Systematic review method expert who works primarily in the field of palliative care.

I confirm that I have read this submission and believe that I have an appropriate level of expertise to confirm that it is of an acceptable scientific standard.
